# Pathologic and biochemical characterization of PrP^Sc^ from elk with *PRNP* polymorphisms at codon 132 after experimental infection with the chronic wasting disease agent

**DOI:** 10.1186/s12917-018-1400-9

**Published:** 2018-03-09

**Authors:** S. Jo Moore, Catherine E. Vrentas, Soyoun Hwang, M. Heather West Greenlee, Eric M. Nicholson, Justin J. Greenlee

**Affiliations:** 1 USDA, Agricultural Research Service, National Animal Disease Center, Virus and Prion Research Unit, Ames, USA; 2 Department of Biomedical Sciences, College of Veterinary Medicine, Iowa State University, Ames, USA

**Keywords:** Chronic wasting disease, Conformational stability, Elk, RT-QuIC, Prion protein

## Abstract

**Background:**

The Rocky Mountain elk (*Cervus elaphus nelsoni*) prion protein gene (*PRNP*) is polymorphic at codon 132, with leucine (L132) and methionine (M132) allelic variants present in the population. In elk experimentally inoculated with the chronic wasting disease (CWD) agent, different incubation periods are associated with *PRNP* genotype: LL132 elk survive the longest, LM132 elk are intermediate, and MM132 elk the shortest. The purpose of this study was to investigate potential mechanisms underlying variations in incubation period in elk of different prion protein genotypes. Elk calves of three *PRNP* genotypes (*n* = 2 MM132, *n* = 2 LM132, *n* = 4 LL132) were orally inoculated with brain homogenate from elk clinically affected with CWD.

**Results:**

Elk with longer incubation periods accumulated relatively less PrP^Sc^ in the brain than elk with shorter incubation periods. PrP^Sc^ accumulation in LM132 and MM132 elk was primarily neuropil-associated while glial-associated immunoreactivity was prominent in LL132 elk. The fibril stability of PrP^Sc^ from MM132 and LM132 elk were similar to each other and less stable than that from LL132 elk. Real-time quaking induced conversion assays (RT-QuIC) revealed differences in the ability of PrP^Sc^ seed from elk of different genotypes to convert recombinant 132 M or 132 L substrate.

**Conclusions:**

This study provides further evidence of the importance of *PRNP* genotype in the pathogenesis of CWD of elk. The longer incubation periods observed in LL132 elk are associated with PrP^Sc^ that is more stable and relatively less abundant at the time of clinical disease. The biochemical properties of PrP^Sc^ from MM132 and LM132 elk are similar to each other and different to PrP^Sc^ from LL132 elk. The shorter incubation periods in MM132 compared to LM132 elk may be the result of genotype-dependent differences in the efficiency of propagation of PrP^Sc^ moieties present in the inoculum. A better understanding of the mechanisms by which the polymorphisms at codon 132 in elk *PRNP* influence disease pathogenesis will help to improve control of CWD in captive and free-ranging elk populations.

## Background

Chronic wasting disease (CWD) is a transmissible spongiform encephalopathy (TSE) that affects a number of cervid species including elk, moose, mule deer, white-tailed deer and reindeer. The TSE’s are a group of neurodegenerative diseases that are characterized by the accumulation of disease-associated prion protein (PrP^Sc^) in the nervous system and other body tissues. In cervids, CWD infection is associated with clinical signs including behavioral abnormalities, excess salivation, emaciation, and eventually death [49].

The host prion protein (PrP) amino acid sequence that is encoded by the prion protein gene (*PRNP*) influences the susceptibility of both humans and animals to TSE’s. Rocky Mountain elk (*Cervus elaphus nelsoni*) are polymorphic at *PRNP* codon 132, encoding either methionine (M) or leucine (L) [30]. The elk *PRNP* codon 132 polymorphism is homologous to the human *PRNP* codon 129 polymorphism that encodes either methionine (M) or valine (V) [39, 40]. In TSE-affected humans, the MM129 genotype is associated with susceptibility to kuru [23] and variant Creutzfeldt-Jakob disease (vCJD) [38]. Some studies have found that elk expressing prion protein homozygous for methionine at codon 132 (hereafter referred to as MM132 elk) are over-represented among CWD-affected elk [11, 12, 31, 41], while another study concluded that elk of all 3 genotypes (MM132, LM132, LL132) show equivalent susceptibility [36]. In experimental studies, LL132 elk orally inoculated with CWD have incubation periods approximately 1.5 times longer than LM132 elk, and 3 times longer than MM132 elk [14, 28]. A better understanding of the biological effects of polymorphisms at elk *PRNP* codon 132 may help to clarify the role of this locus in the spread of CWD in North American elk populations.

Here, we provide further histopathologic characterization of experimental CWD infection in MM132, LM132 [14] and LL132 [28] elk. We examine the intersection of host genotype, incubation period, PrP^Sc^ fibril stability, and amyloid formation rate and demonstrate that genotype-dependent differences in PrP^Sc^ stability and amyloid formation rate may contribute to the observed variation in incubation periods of elk of different genotypes. These results may help us to better understand the influence of the *PRNP* 129 polymorphism in human prion diseases.

## Methods

### Ethics statement

This experiment was carried out in accordance with the Guide for the Care and Use of Laboratory Animals (Institute of Laboratory Animal Resources, National Academy of Sciences, Washington, DC) and the Guide for the Care and Use of Agricultural Animals in Research and Teaching (Federation of Animal Science Societies, Champaign, IL). The Institutional Animal Care and Use Committee at the National Animal Disease Center reviewed and approved the animal use protocol (protocol number: 3833).

### Inoculum preparation and animal procedures

The source, genotyping, husbandry and oral inoculation of the eight elk in this study has been described previously [14]. Briefly, elk were obtained from a captive elk game farm on which 79 cases of CWD were diagnosed between 1997 and 2001. All CWD-positive elk were of the MM132 or LM132 genotypes; no cases were found in LL132 elk [28]. MM132 and LM132 elk calves were sourced from the 2000 birth cohort of 1 of the 3 premises operated by the captive elk game farm; LL132 elk calves were sourced from the 2001 birth cohort of a different premises to the MM132 and LM132 calves. Genotype analysis was conducted on nucleic acid extracted from live animal blood samples as described previously [8]. The inoculum was prepared from pooled brain material from one MM132 and one LM132 elk (equal parts MM132 and LM132 donor tissue), both of which had showed clinical signs of CWD. At 8 months of age four LL132 elk, two LM132 elk and two MM132 elk were inoculated orally with 3 mL of inoculum daily for five consecutive days (total dose equivalent to 15 g of pooled brain) [14, 28]. Elk were housed in a biosafety level-2 isolation barn at the National Animal Disease Center (Ames, IA). This barn had not previously housed CWD-infected animals and entry and exit procedures were in place to eliminate potential cross-contamination from any source. Health was monitored twice daily. Sentinel LL132 animals were not included in the study design.

Animals were necropsied after being found dead, or euthanized upon showing clinical signs or at the conclusion of the experiment at 64 months postinoculation (MPI). Two sets of tissue samples were collected. One set of tissues included representative sections of: brain, eye (retina), optic nerve, sciatic nerve, trigeminal ganglion, peripheral nerves (optic, sciatic), lymph nodes (retropharyngeal, mesenteric, popliteal, prescapular), tonsils (palatine, pharyngeal), 3rd eyelid, foregut (esophagus, reticulum, omasum, rumen, abomasum), jejunum, ileum, recto-anal mucosa-associated lymphoid tissue (RAMALT), salivary gland, liver, pancreas, kidney, urinary bladder, spleen, adrenal, pituitary, thyroid, skeletal muscles (diaphragm, biceps femoris, masseter, psoas major, triceps), heart muscle, tongue, turbinate, lung, trachea, skin. These tissues were fixed in 10% buffered formalin, embedded in paraffin wax, and sectioned at 5 μm for microscopy examination after hematoxylin and eosin staining. The second set of tissues, comprising subsamples of all tissues collected into formalin, was frozen.

### Immunohistochemistry

All paraffin-embedded tissues were immunostained by an automated immunohistochemical method for detection of PrP^Sc^ as described previously [9] using the anti-PrP monoclonal antibody F99/96.7.1 [29].

### Antigen-capture enzyme immunoassay (EIA)

The IDEXX HerdChek BSE-Scrapie Ag EIA plate (Westbrook, ME) was used with modifications for the EIA-based fibril stability assay and the determination of PrP^Sc^ levels. Brain samples from elk were recovered from archived frozen brainstem stored at either − 20 °C or − 80 °C. Brainstem samples were mixed with 1X PBS (phosphate-buffered saline, lacking calcium and magnesium) and homogenized in a bead beater.

### EIA-based fibril stability assay

PrP^Sc^ fibril stability was determined using an EIA-based assay as described previously ([9]. This assay is a protease-free method to monitor PrP^Sc^ unfolding that exposes the epitopes for the antibodies used in the IDEXX assay. The capture surface of the IDEXX EIA is a proprietary ligand that is specific for misfolded protein with detection of bound protein by a PrP specific antibody, and does not require protease digestion to distinguish PrP^Sc^ from PrP^C^. Briefly, dilutions of elk brain samples were incubated at concentrations of guanidine hydrochloride (GdnHCl) over a range from 0.25 M to 4.0 M. Neither brainstem samples nor intact brain were available for MM132 elk #2 so spinal cord was used for a comparison of elk #2 and elk #1; sections of gray matter from the cervical spinal cord were excised and homogenized as for the brainstem samples. The relative amount of PrP^Sc^ remaining was assessed by the EIA optical density (OD_450_) after dilution of treated brain homogenates to a final [GdnHCl] of 0.25 M and application to the IDEXX plate. The amount of PrP^Sc^ remaining was then plotted against GdnHCl concentration. The midpoint of the curve, or [GdnHCl]_1/2_, is defined as the concentration of GdnHCl at which the PrP^Sc^ signal was reduced by half of the signal at 0.25 M GdnHCl; PrP^Sc^ with a smaller [GdnHCl]_1/2_ is less stable. As described previously [44], due to variations in the upper baseline shape, the Smooth Line function in Microsoft Excel was used to connect data points in each curve and visualize the midpoint.

### Calculation of amount of PrP^Sc^ versus incubation period

To determine the relative amount of PrP^Sc^ in brain from elk at clinical disease, 1% *w*/*v* brain homogenates were serially diluted in 1X PBS and tested using the EIA assay and diluted until the OD_450_ readings were in the linear range of detection. To provide a normalization metric across multiple samples, the 1% (*w*/*v*) homogenate was assigned a brain unit equivalent (BU) value of 100 and equivalent BU’s were calculated for each dilution, i.e. 1:2 dilution = 50 BU, 1:4 dilution = 25 BU. For each sample, the EIA OD reading in the linear range (minus the negative control value) was divided by the BU of the dilution at which the linear range OD was measured, to generate an OD/BU value. We then calculated the ratio of the OD/BU values for each sample compared to the sample with the lowest OD/BU value. Ratio values were plotted against incubation period.

### Recombinant prion protein production and purification

*E. coli* (BL21(λDE3)) was transformed with the pET28a vector containing the elk PrP gene corresponding to mature length PrP (amino acids 23–231, GenBank accession number AAC12860.2), and elk recombinant PrP was expressed and purified as described for bovine PrP [17, 46]. The concentration of filtered protein eluent was determined by UV absorbance at 280 nm using an extinction coefficient of 59,485 M^− 1^ cm^− 1^ as calculated for mature length elk prion protein.

### Real-time quaking induced conversion (RT-QuIC) protocol

RT- QuIC was performed on 10% (*w*/*v*) brainstem homogenized in PBS from elk #1 (MM132), elk #4 (LM132) and elk #7 (LL132) as described previously [17]. The reaction mix was composed of 10 mM phosphate buffer (pH 7.4), 400 mM NaCl, 0.1 mg/ml recombinant mature length elk prion protein (132 L, [23–231]; 132 M, [23–231]), 10 μM thioflavin T (ThT), 1 mM ethylenediaminetetraacetic acid tetrasodium salt (EDTA). The positive threshold was calculated as the mean value of normal elk brain homogenates plus 10 standard deviations. Previously described criteria were applied for classification of positive samples of RT-QuIC [5, 32, 34].

## Results

### Differences in incubation period were associated with polymorphisms at *PRNP* codon 132

At approximately 23 MPI MM132 elk (animals #1 and #2) developed loss of appetite and body condition. Both elk rapidly became unable to stand without assistance and were euthanized. At 38 (#3) and 40 (#4) MPI respectively, LM132 elk developed similar clinical signs and were euthanized (average incubation period = 39 MPI) (Table 1). The first LL132 elk (#5) to succumb to CWD was found dead at 59 MPI. This elk had previously been noted to be smaller and thinner than the other LL132 elk. During month 63 post-inoculation elk #6 developed muscle fasciculations, staggering, tremor, anorexia, mental dullness, head pressing and loss of bladder control, and was euthanized. The two remaining elk (#7 and #8) were euthanized at 64 MPI after displaying early signs of clinical disease, including subtle behavior changes, mild loss of body condition, and roughened hair coat (Table 1). The average incubation period for the four LL132 elk was 62.8 MPI.

**Table 1:** Animal information and results for study elk

Animal number	1	2	3	4	5	6	7	8
Genotype codon 132	MM	MM	LM	LM	LL	LL	LL	LL
Incubation period (MPI)	23	23	38	40	60	63	64	64
Clinical presentation	LBC, Rec	LBC, Rec	LBC, ataxia	LBC	LBC	Neuro	FD	LBC
Tissue results								
Brain	+/+	+/+	+/+	+/+	+/+	+/+	+/+	+/+
Retina	+	+	+	+	+	+	+	+
Peripheral NS	+	+	+	–	+	+	–	+
Lymphoid head	+	+	+	+	+	+	+	+
Lymphoid other	+	+	+	+	+	+	+	n/a
Intestines	+	+	+	+	–	+	+	+
Spleen	+	+	+	–	+	–	–	+
Pituitary	n/a	n/a	+	+	n/a	+	–	n/a
Foregut	n/a	–	–	+	–	–	–	+
Adrenal	n/a	+	–	–	n/a	–	+	n/a

*M* methionine, *L* leucine, *MPI* months post inoculation, *LBC* Loss of body condition, *Rec* recumbency, *Neuro* neurological signs (for more detail see Results), *FD* found dead. Tissue results: brain, vacuolation/PrP^Sc^; other tissues, PrP^Sc^; n/a, tissue not available for examination

### Spongiform change was more prominent in the gray matter in MM132 and LM132 elk, while in LL132 elk the white matter was more severely affected

**Fig. 1:**
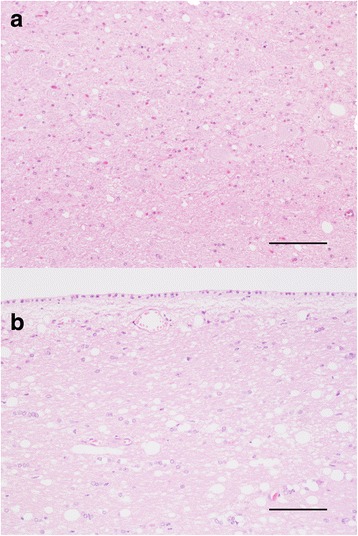
Spongiform change observed in elk inoculated with the CWD agent. **a** Spongiform change in the dorsal motor nucleus of the vagus nerve in elk #2 (MM132). (Hematoxylin and eosin, original magnification 20×). **b** White matter vacuolation in the corpus callosum in elk #8 (LL132). (Hematoxylin and eosin, original magnification 10×)

To investigate the patterns of spongiform change in the brain, hematoxylin and eosin stained coronal sections of brain and spinal cord were examined by light microscopy. Pathologic changes in MM132 and LM132 elk have been described previously [14]. Microscopic lesions of spongiform encephalopathy were present in all elk. In LM132 and MM132 elk, microcavitation of the gray matter was more prevalent than intraneuronal vacuolation and neuronal degeneration, and there was mild astrocytosis [14]. In all LM132 and MM132 elk, moderate to severe spongiform change was present in the dorsal motor nucleus of the vagus nerve (Fig. 1a) and surrounding nuclei. In LL132 elk, vacuolation of white matter tracts (Fig. 1b) was more prevalent than microcavitation of the gray matter.

In summary, microcavitation of gray and white matter was observed in all elk. Spongiform change was more prominent in the gray matter of LM132 and MM132 elk and more prominent in the white matter of LL132 elk.

### PrP^Sc^ accumulation in LM132 and MM132 elk was primarily neuropil-associated while intra-glial immunoreactivity was prominent in LL132 elk

To investigate the patterns of PrP^Sc^ deposition in the brain, immunolabeled sections of brain, spinal cord, and peripheral tissues were examined by light microscopy. Subjectively, the total amount of PrPSc immunoreactivity was greater in MM132 and LM132 elk compared to LL132 elk. In LM132 and MM132 elk, PrP^Sc^ immunoreactivity in the brain appeared as coarse granular material that was scattered throughout the neuropil. Perineuronal immunolabeling was common while intraneuronal immunolabeling was rare [14].

**Fig. 2:**
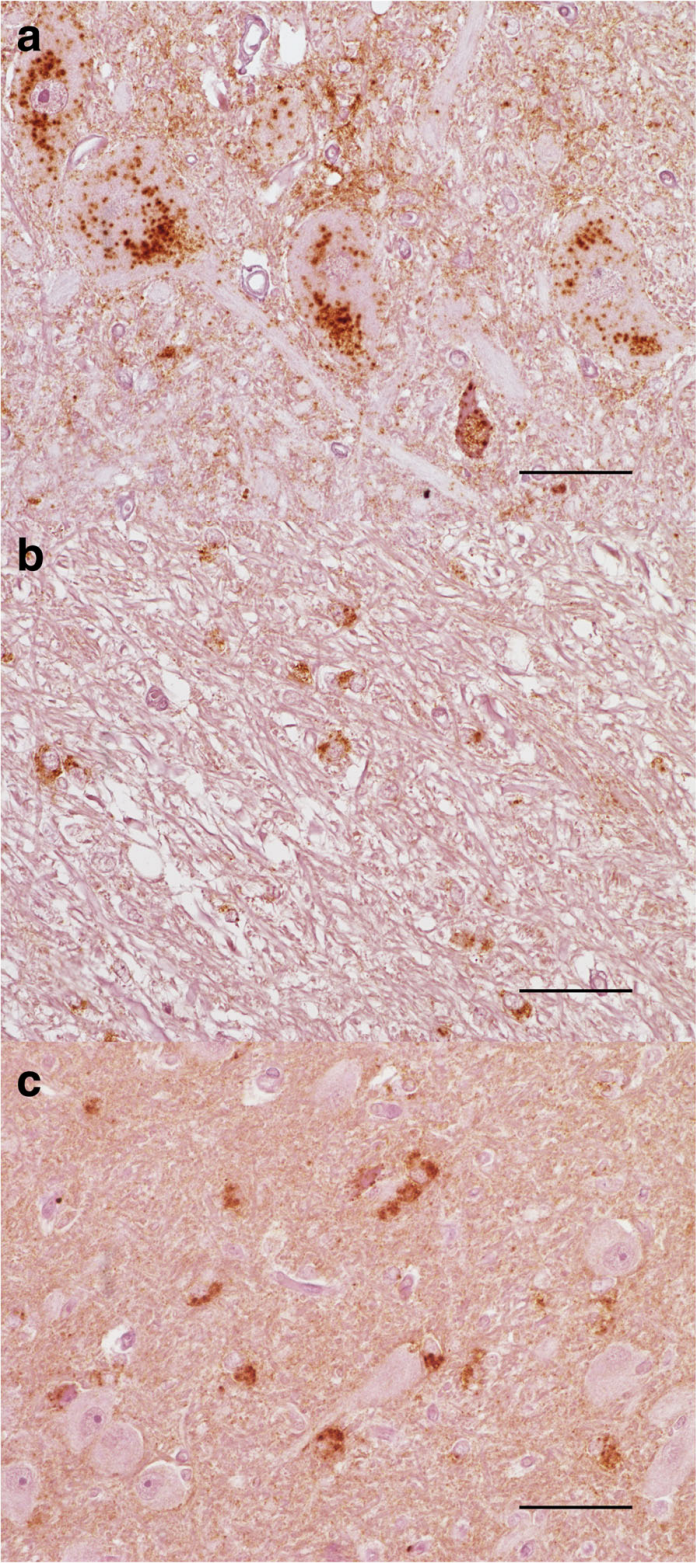
Spongiform change and patterns of PrP^Sc^ immunoreactivity observed in elk inoculated with the CWD agent. **a** Intraneuronal immunoreactivity in the hypoglossal nucleus in elk #5 (LL132) elk. **b** Glial associated immunoreactivity in the cerebellar white matter of elk #5 (LL132). **c** Glial associated immunoreactivity in the lateral geniculate nucleus of elk #5 (LL132). **a**-**c** immunostained using monoclonal anti-PrP antibody F99/96.7.1, original magnification 40×

In LL132 elk, coarse granular and perineuronal immunolabeling were common, as was intraneuronal immunolabeling (Fig. 2a). In addition, there was granular to punctate immunolabeling that was often associated with astrocytes. This astrocyte-associated immunolabeling was most prominent in white matter (Fig. 2b) but also was observed in gray matter (Fig. 2c).

In elk of all genotypes, PrP^Sc^ was abundant in the lymphoid follicles of the palatine tonsil, retropharyngeal lymph node and gut-associated lymphoid tissue. The skeletal muscles (M. biceps femoris, M. masseter, M. psoas major, M. triceps), diaphragm, kidney, urinary bladder, nose skin, turbinate, trachea, lung, tongue, liver, pancreas, salivary gland, and thyroid were negative in all samples examined.

PrP^Sc^ immunoreactivity was widespread in the central nervous system and peripheral lymphoid tissues of all elk. Intraneuronal immunolabeling was less prominent in LM132 and MM132 elk compared to LL132 elk. Glial-associated immunolabeling observed in LL132 elk was not seen in LM132 or MM132 elk.

### PrP^Sc^ fibrils from LL132 elk are more stable than fibrils from LM132 and MM132 elk

To determine whether there is an association between fibril stability of PrP^Sc^ and incubation period in CWD-affected elk, we assessed the stability of PrP^Sc^ using an EIA-based stability assay.

**Fig. 3:**
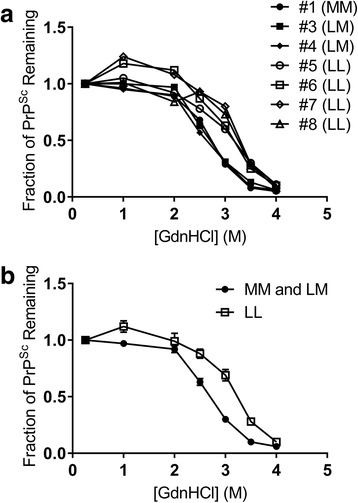
The fibril stability of PrP^Sc^ from MM132 and LM132 elk was lower than the fibril stability of PrP^Sc^ from LL132 elk. Homogenates of infected elk brain were incubated in GdnHCl at the indicated concentration as described in Methods, with remaining PrP^Sc^, as detected by EIA, expressed as a fraction of the signal after treatment with 0.25 M GdnHCl. **a** Comparison of individual animals of MM132, LM132 and LL132 genotype elk, as indicated. Data were averaged across 4–6 technical replicates to generate each curve. **b** Average curves for LL132 elk (closed symbols) as compared to MM132 and LM132 elk (open symbols) from (**a**); error bars depict +/− the standard error of the mean (SEM) of the biological replicates

When the fibril stability of PrP^Sc^ in homogenized brainstem of elk of each genotype was measured, two clusters of curves were evident (Fig. 3a). Samples from MM132 and LM132 elk exhibited lower fibril stability, with a [GdnHCl]_1/2_ of ≈2.75, while samples from LL132 elk exhibited higher fibril stability, with a [GdnHCl]_1/2_ of ≈3.2–3.3. When fibril stability data from samples from MM132 and LM132 elk are combined and compared to LL132 elk samples (Fig. 3b), average values of LL132 versus M132-containing groups (MM132 and LM132) exhibited statistically significant differences at 2.5, 3, and 3.5 M GdnHCl (*p* < 0.004, t-test with unequal variances). Since unfixed brainstem tissue was unavailable for the second MM132 elk (elk #2) spinal cord homogenate was used to determine the fibril stability of PrP^Sc^ from this elk. The stability of PrP^Sc^ from the elk #2 spinal cord sample was similar to PrP^Sc^ from brainstem homogenate from the other MM132 elk (#1) in the study (data not shown).

In summary, PrP^Sc^ from samples from MM132 and LM132 elk that have short and intermediate incubation periods, respectively, was less stable than PrP^Sc^ from samples from LL132 elk that have the longest incubation periods.

### Relative amount of PrP^Sc^ in comparison to incubation period in elk

To investigate the relationship between the incubation period and relative amount of PrP^Sc^ accumulation in the brain, the amount of PrP^Sc^ in brain homogenates was quantified using EIA.

**Fig. 4:**
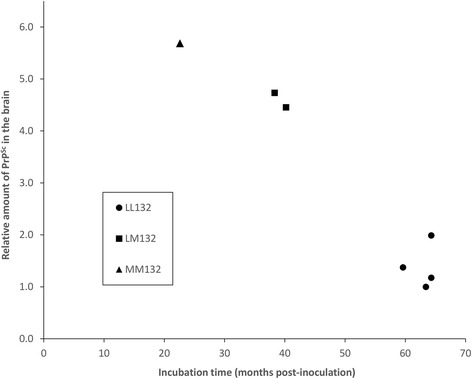
The relative amount of PrP^Sc^ in the brainstem (obex) is strongly associated with incubation period. The amount of PrP^Sc^ in the brain was calculated using optical density readings from an antigen-capture enzyme immunoassay (EIA). The relative amount of PrP^Sc^ in the brain of the elk with the lowest EIA result in the linear range was designated a baseline value of 1.0. Results for other elk are expressed as a ratio relative to the baseline elk. Frozen obex was not available for elk #2 (MM132) so this animal is not included in the figure

The relative amount of PrP^Sc^ in the brain was lowest for LL132 elk, intermediate for LM132 elk, and highest for MM132 elk. When the relative amount of PrP^Sc^ in the brain was plotted against elk incubation period, a strong negative correlation between these two variables was apparent (Fig. 4).

### Real-time quaking induced conversion assays seeded with samples from LM132 and MM132 elk produced shorter lags times in 132 M substrate and longer lag times in 132 L substrate

To investigate if RT-QuIC can be used to detect differences in either conversion efficiency of the substrate or the prion seeding activity from CWD infected elk brain of different genotypes, we used infected and normal elk brain homogenates as seed for mature length recombinant 132 L or 132 M elk prion protein substrates. To allow for comparison between substrates, all assays were performed in the same reaction conditions as described in the Materials and Methods.

**Fig. 5:**
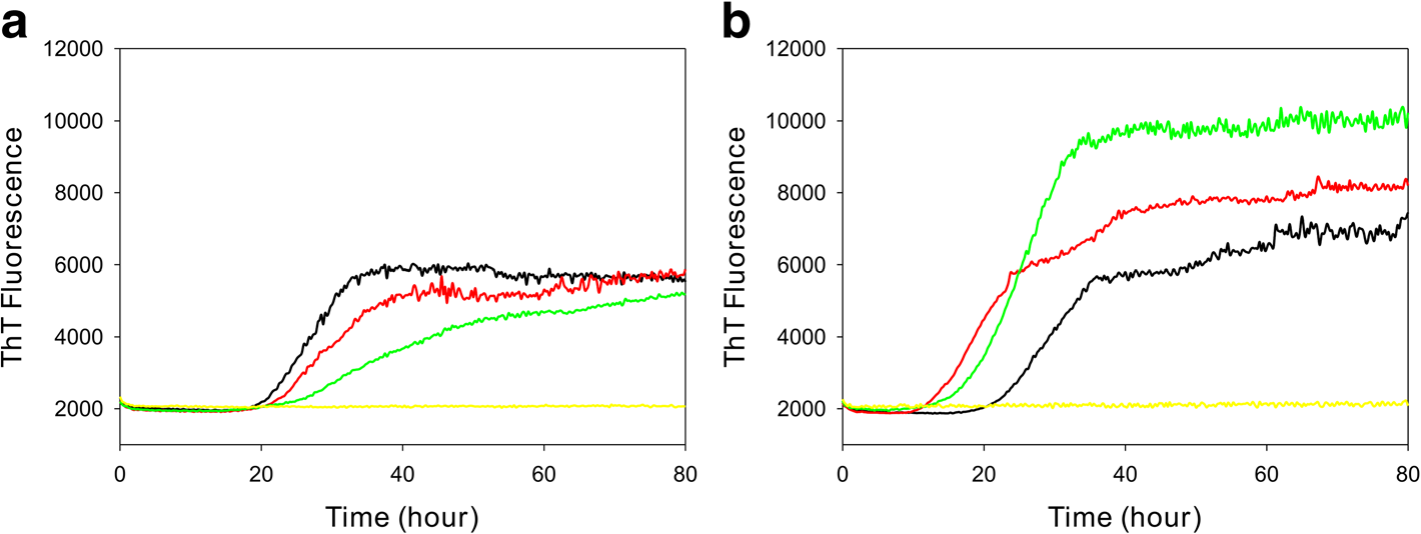
RT-QuIC detection of seeding activity in elk brain samples using mature length recombinant elk prion protein 132 L (**a**) and 132 M (**b**) substrates. RT-QuIC assays seeded with MM132 (green), LM132 (red), LL132 (black) or negative seed (yellow) are shown. RT-QuIC reaction mixtures were seeded with 10^− 2^ dilutions of normalized brain homogenate. A final 400 mM NaCl was used with each substrate. Data are presented as mean ThT fluorescence of quadruplicate reactions

Using the 132 L substrate (Fig. 5a) and 132 M substrate (Fig. 5b) an increase in Thioflavin-T fluorescence, indicating the presence of misfolded prion protein, was observed in each quadruplicate reaction seeded with 10^− 2^ dilution of normalized elk brain homogenate, but no increase in fluorescence was observed in reactions seeded with normal brain homogenates. The lag times in assays using the 132 L substrate were similar for seeds from elk of all three genotypes (LM132 = 21 h, MM132 = 23 h, LL132 = 20.5 h). For LM132 and MM132 seeds, the lag times in assays using 132 M substrate (LM132 = 12 h, MM132 = 12.5 h) were shorter than the lag times in assays using 132 L substrate (LM132 = 21 h, MM132 = 23 h). The lag time for the LL132 seed in 132 L substrate (19 h) was similar to the lag time in 132 M substrate (20.5 h).

## Discussion

We demonstrate that the shorter incubation periods of elk that are homozygous for methionine at *PRNP* codon 132 (MM132) or heterozygous for leucine and methionine (LM132) elk are associated with PrP^Sc^ that is less stable than PrP^Sc^ from elk that are homozygous for leucine (LL132), which have the longest incubation periods. Subjectively, the amount of PrP^Sc^ immunoreactivity in the brain was similar across elk of all genotypes using IHC. However, serial dilution studies using EIA revealed that the brains of LL132 elk contain relatively lower amounts of PrP^Sc^ than LM132 and MM132 elk. Although the interpretation of results from this study is limited by the small number of elk of each genotype that were available for inoculation, this study nevertheless provides valuable baseline data on the relationship between *PRNP* codon 132 genotype and disease pathogenesis in elk with chronic wasting disease.

We observed a strong negative association between incubation period and the relative amount of PrP^Sc^ in the brain in elk of different genotypes, i.e. elk with longer incubation periods accumulate less PrP^Sc^. Differences in the relative amount of PrP^Sc^ in the brain were detected using EIA on frozen brain tissue and IHC on formalin-fixed paraffin-embedded brain tissue. This suggests that MM132 elk may be more permissive to PrP^Sc^ accumulation than LM132 elk. This observation is supported by a previous study in transgenic mice that showed that the L132 polymorphism severely restricts propagation of CWD prions [7]. Since PrP^Sc^ from MM132 and LM132 elk show similar fibril stability profiles and RT-QuIC conversion profiles using recombinant 132 L and 132 M elk prion protein, one explanation for the rapid PrP^Sc^ accumulation in MM132 elk may be a potential difference in the effective concentration of PrP^C-132M^. In heterozygous sheep, both allelic variants of PrP^C^ are present in equal amounts [27]. It is assumed that this relationship is similar in heterozygous elk, which means that the amount of PrP^C-132M^ in MM132 elk is twice that of LM132 elk. In transgenic mice higher expression levels of PrP^C^ result in reduced incubation times (reviewed in [47]). Therefore, the relatively higher proportion of PrP^C-132M^ in MM132 elk compared to LM132 elk may contribute to the relatively shorter incubation times observed in MM132 elk.

In sheep, conversion of PrP^C^ to PrP^Sc^ is more efficient when the *PRNP* genotype of the inoculum and substrate are the same [2, 3, 21]. Furthermore, in heterozygous animals there is preferential conversion of the PrP^C^ moiety of the allele associated with a higher susceptibility to disease [18, 27]. Since the biological behavior of scrapie prions in sheep and CWD prions in cervids are similar, it seems reasonable to assume that the conversion efficiency of elk CWD prions has a sequence dependence similar to sheep scrapie prions. The brain homogenate used to inoculate the elk was prepared from pooled brain material from one MM132 and one LM132 elk. Titration of brain homogenate was not performed prior to pooling. Based on observations in sheep [18, 27] it is probable that the PrP^Sc^ in the LM132 brain was predominantly PrP^Sc-132M^ and therefore that the pooled brain homogenate contained mostly PrP^Sc-132M^. This PrP^Sc-132M^ would propagate more efficiently in elk expressing PrP^C-132M^ than those expressing PrP^C-132L^ or a mixture of PrP^C-132M^ and PrP^C-132L^. Experimental challenge of elk of each genotype with brain homogenates from homozygous and heterozygous donors may help to elucidate the relative contribution of donor and recipient *PRNP* genotypes to incubation time in CWD-affected elk.

If relative incubation period reflects the relative permissibility of elk of different *PRNP* genotypes to PrP^Sc^ accumulation and by extension, their susceptibility to disease, our findings support previous CWD surveys that have shown that MM132 elk are most susceptible to CWD, the susceptibility of LM132 elk is intermediate, and LL132 elk are least susceptible to CWD [31, 41]. These findings suggest that genetic selection for the L132 allele has the potential to reduce the impact of CWD in captive and free-ranging elk populations, although it should be kept in mind that the protective effects of the L132 allele against CWD prions are not absolute [4, 7]. The elk breeding facility from which the elk calves for this experiment were obtained was known to have a high prevalence of CWD [14] so infection of elk calves with CWD prior to being moved to the quarantine facility at 8 months of age cannot be ruled out. However, since incubation periods for elk within each genotype group were similar to each other and different to elk of different genotypes, it appears that potential infection at the breeding facility did not influence the outcome of experimental infection at the quarantine facility in this study.

We have shown that the fibril stability of PrP^Sc^ from elk with shorter incubation periods (i.e. MM132 and LM132) is lower, while PrP^Sc^ fibrils from elk with longer incubation periods (LL132) are more stable. These observations are in agreement with previous observations in mice challenged with synthetic [24] and mouse-adapted [1, 25] prion strains, and in sheep challenged with different scrapie isolates [45]. It is hypothesized that lower fibril stability leads to increased PrP^Sc^ fibril fragmentation that facilitates the conversion of PrP^C^ to PrP^Sc^ and results in faster replication of PrP^Sc^ and reduced incubation periods [42, 51]. However, an inverse relationship between incubation period and fibril stability – that is, PrP^Sc^ from animals with shorter incubation periods is more stable – has been observed in Syrian hamsters challenged with hamster adapted scrapie or transmissible mink encephalopathy strains [35], sheep with naturally occurring classical or Nor98 scrapie [37, 48], and cattle challenged with classical or atypical (H-type) bovine spongiform encephalopathy [44]. These variable relationships between incubation period and fibril stability suggest that factors other than, or in addition to, fibril stability of PrP^Sc^ can influence incubation periods.

Western blot analyses of brain samples from elk in this study have been published previously [14, 28]. The three characteristic bands of the proteinase-resistant core of PrP^Sc^ were observed in all elk and samples from MM132 and LM132 elk showed similar migration profiles, glycoform ratios, and N-terminal cleavage sites [14, 28]. However, samples from LL132 elk showed a significantly lower mean apparent molecular mass compared to MM132 and LM132 elk; this was associated with cleavage near residues 98–113 [28], as compared to cleavage at residues 78 and 82 in MM132 elk [50]. Therefore, similar to fibril stability and amyloid formation rate, western blot phenotype does appear to be a strongly associated with differences in incubation periods in MM132 and LM132 elk.

Until now, RT-QuIC applications in cervids have mainly focused on detection of small amounts of prions in fluids and tissues relevant to pre-clinical diagnosis of disease or disease transmission: urine [15, 20], feces [4, 20], saliva [15, 16], blood [6], cerebrospinal fluid [13], rectal biopsy and nasal brush samples [11, 12]. RT-QuIC has also been utilized for the discrimination of subtypes of bovine spongiform encephalopathy (BSE) [17, 26, 33].

To investigate if conversion efficiency of PrP^Sc^ influences incubation period, real-time quaking induced conversion (RT-QuIC) was performed using recombinant mature length elk prion protein (132 L and 132 M) seeded with brain homogenates from one elk of each genotype. These experiments revealed differences in the ability of PrP^Sc^ seed from CWD-infected elk of different genotypes to convert recombinant elk prion substrate. The MM132 or LM132 seeds convert 132 M substrate protein readily, whereas LL132 seed is much slower to do so. In contrast, all seeds convert 132 L substrate protein although the LL132 seed exhibited the fastest conversion. This conversion data suggests two potential hypotheses: (a) there are two distinct and stably propagating conformations of elk PrP^Sc^ present, one that is adopted more readily by 132 M protein and one that is adopted more readily by 132 L protein; or (b) the differences in conversion rate (both in the animal and in RT-QuIC) are the result of genotype mismatches between seed PrP^Sc^ and substrate. The similar lag phases observed with MM132 and LM132 seed are consistent with previously reported RT-QuIC analyses [10] and the fibril stability results reported here, and may provide further evidence that the LM132 seed contains a relatively large proportion of PrP^Sc-132M^. The results of the stability assay also provide evidence that there are two conformations with distinct molecular properties, but future investigations are needed to explore this question. Inoculation of both the MM132 and LL132 seeds into transgenic mice carrying the elk prion gene will be useful in assessing differences in PrP^Sc^ fibril stability and incubation times upon serial passage into mice of a single *PRNP* genotype.

PrP^Sc^ from CWD-infected LL132 elk shares a number of immunohistochemical features with the ovine scrapie strain CH1641, namely a loss of the epitope for the anti-PrP monoclonal antibody P4 that binds PrP residues 93–99 [43], and reduced but detectable immunoreactivity with the monoclonal antibody 8G8 (that binds residues 98–113 [22]) [19, 28]. The phenotype of PrP^Sc^ accumulation in the brain of sheep with CH1641 is characterized by prominent intracellular immunoreactivity in neurons and glial cells, and relatively little extracellular immunoreactivity [19]. Intraneuronal PrP^Sc^ accumulation is rare in MM132 and LM132 elk with CWD [14] but was commonly observed in the LL132 elk in this study. Furthermore, glial-associated immunolabeling was prominent in LL132 elk and not observed in MM132 or LM132 elk.

## Conclusions

This study provides further evidence of the importance of *PRNP* genotype in the pathogenesis of CWD of elk. We have shown that the biochemical properties of PrP^Sc^ from MM132 and LM132 elk are similar to each other and different to PrP^Sc^ from LL132 elk. The shorter incubation periods in MM132 compared to LM132 elk may be the result of genotype-dependent differences in the efficiency of propagation of PrP^Sc^ moieties present in the inoculum. Further work is needed to develop a better understanding of the underlying mechanisms by which the polymorphisms at codon 132 in elk *PRNP* influence disease pathogenesis, with a view to improving control of CWD in captive and free-ranging elk populations.

## Abbreviations

CWD: Chronic wasting disease
EIA: Antigen-capture enzyme immunoassay
MPI: Months postinoculation
*PRNP*: Prion protein gene
PrP^C^: Cellular prion protein, not associated with disease
PrP^Sc^: Disease-associated prion protein
RAMALT: Recto-anal lymphoid tissue
RT-QuIC: Real-time quaking induced conversion
TSE: Transmissible spongiform encephalopathy
vCJD: variant Creutzfeldt-Jakob disease
